# Climate change as a driver of food insecurity in the 2007 Lesotho-South Africa drought

**DOI:** 10.1038/s41598-021-83375-x

**Published:** 2021-02-16

**Authors:** Jasper Verschuur, Sihan Li, Piotr Wolski, Friederike E. L. Otto

**Affiliations:** 1 Environmental Change Institute, University of Oxford, Oxford, UK; 2 Oxford E-Research Centre, Department of Engineering Science, University of Oxford, Oxford, UK; 3 Climate System Analysis Group, University of Cape Town, Cape Town, South Africa

**Keywords:** Climate change, Environmental impact

## Abstract

Climate-induced food production shocks, like droughts, can cause food shortages and price spikes, leading to food insecurity. In 2007, a synchronous crop failure in Lesotho and South Africa—Lesotho’s sole trading partner—led to a period of severe food insecurity in Lesotho. Here, we use extreme event attribution to assess the role of climate change in exacerbating this drought, going on to evaluate sensitivity of synchronous crop failures to climate change and its implications for food security in Lesotho. Climate change was found to be a critical driver that led to the 2007 crisis in Lesotho, aggravating an ongoing decline in food production in the country. We show how a fragile agricultural system in combination with a large trade-dependency on a climatically connected trading partner can lead to a nonlinear response to climate change, which is essential information for building a climate-resilient food-supply system now and in the future.

## Introduction

Globally, two billion people are subject to moderate to severe food insecurity^1^. Climate variability accounts for approximately 30% of the variability in global agricultural yields^2^, in turn increasing the uncertainty of food production and prices across various geographical scales, threatening food security^3,4,5,6^. Climate-induced risks to food insecurity are driven by the exposure to climate extremes (e.g. extreme droughts) and the vulnerability and response of the food-supply system to production shocks^6–8^. The impacts of reduced production and price spikes are often felt disproportionately by poor consumers, who spend a larger share of their household budget on staple foods^5^, potentially leading to them foregoing consumption or being pushed into poverty^9^.

Previous work has identified the geographical distribution of climate impacts on agricultural yields^4,6,10,11^. For adaptation purposes, however, it is important to evaluate how climate change (CC) compares to other drivers of food insecurity across various temporal and spatial scales. In particular, characteristics of local and regional food systems, such as the presence of irrigation, trade dependencies, crop diversity and socio-demographic factors will determine to what extent climate shocks will ultimately affect food security in a certain region. Understanding the influence of CC in past events that caused food insecurities can help identify the response of the food system to CC and shape adaptation strategies in countries more vulnerable to climate-induced food insecurities.

Here, we lay out a methodology in which an extreme event attribution (EEA)^12,13^ approach is combined with an exploratory modelling framework to extend the evaluation of the role of CC from hazard to food security. We apply this methodology to the 2007 drought in Lesotho and South Africa that led to a synchronous maize production failure, requiring emergency assistance for 400,000 people in Lesotho (~ 20% population)^14^. We illustrate that Lesotho’s vulnerability to food insecurity shows a nonlinear response to CC, which is essential information to consider in building a climate-resilient food-supply system now and in the future.

## The 2007 drought

We focus our analysis on maize production in both countries, as maize is the main staple food in Lesotho, constituting 77% of agricultural production and 80% of the rural diet^14^. During normal years, around 30% of the domestic demand for maize is satisfied by national production, with the remaining being imported duty-free from South Africa through the Southern African Customs Union free trading zone.

**Figure 1:**
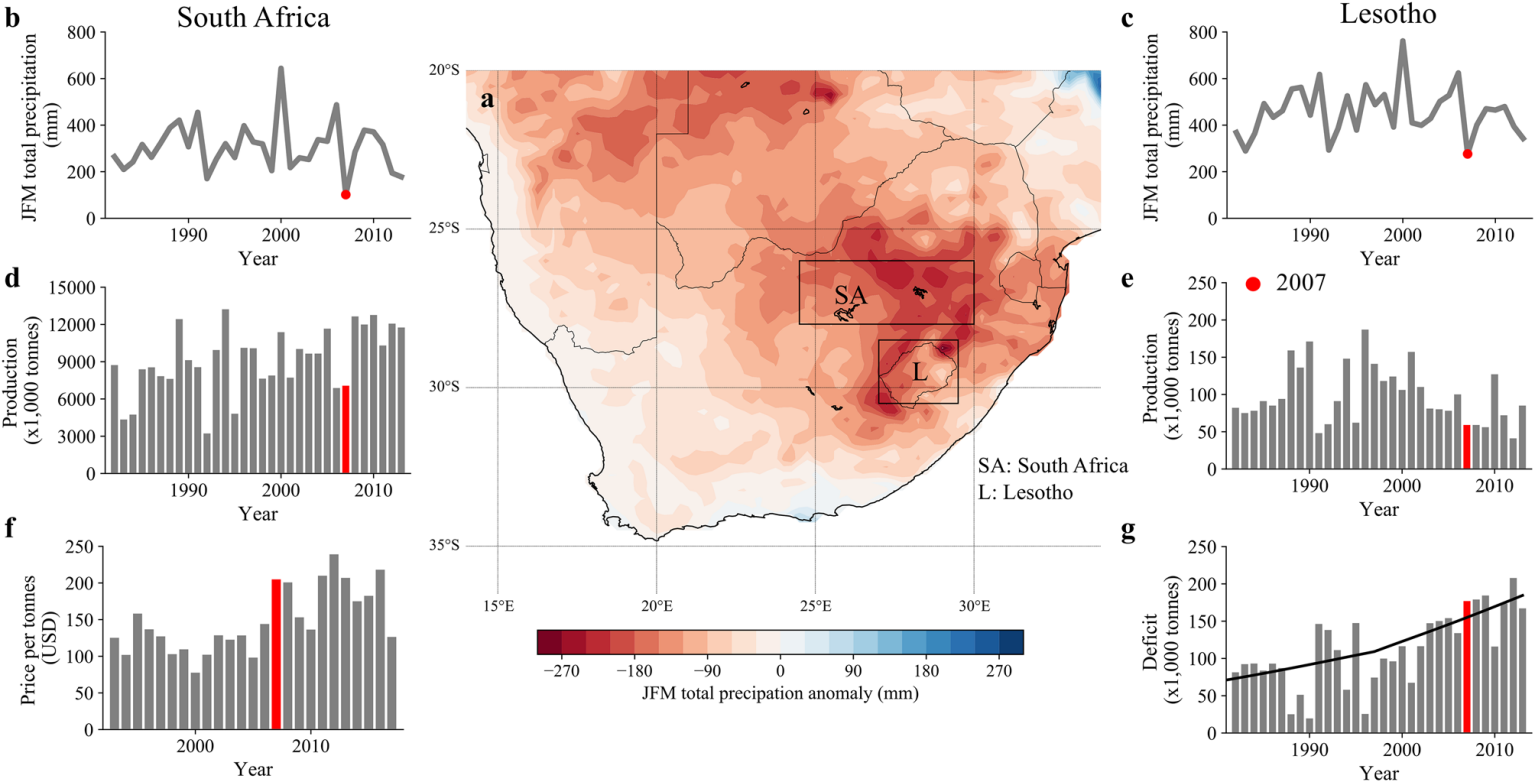
Overview of 2007 drought and impacts. (**a**) Spatial distribution of the JFM precipitation anomaly over South Africa (relative to 1979–2018 average) derived from reanalysis data^17^, with the boxes indicating the areas that the climate data is averaged over. (**b**) JFM precipitation time series for South Africa with the 2007 event highlighted in red. (**c**) Same as (**b**) but for Lesotho. (**d**) Maize production data from the FAOSTAT database^18^ with the 2007 event in red. (**e**) Same as (**d**) but for Lesotho. (**f**) Price per tonnes for maize in South Africa from FAOSTAT. Note only data from 1991 onwards is available. (**g**) Maize deficit (minimum maize demand for sufficient calorie intake minus domestic production) over time in Lesotho, with the black line showing the non-linear trend line over the years, derived using a lowess function. Figure (**a**) was generated using the ‘Basemap’ package (https://matplotlib.org/basemap/index.html) and Python Programming Language (version 3.7).

The 2007 drought was characterised by anomalously low precipitation (compared to 1979–2018) over the eastern part of South Africa and Lesotho (Fig. 1a). during the agricultural growing season (January–February–March, JFM)^14,15^. Although temperature was also anomalously high (1 in 5-year event), we focus on JFM total precipitation anomalies as the dominant climatic driver of maize yield during this event given the severity of the precipitation anomaly and the predominant rainfed agriculture in this region^14–16^. We averaged precipitation data over Lesotho and the main maize growing region of South Africa (boxes in Fig. 1a). From reanalysis data (ERA5^17^) over 1979–2018 (see “Methods”), we estimate the 2007 event to be the most extreme JFM total precipitation deficit on record in both countries (Fig. 1b,c), resulting in the most severe co-occurring drought on record (Fig. S1a). Figure 1d and e show the maize production time series (FAOSTAT^18^) in both countries, and Fig. 1g shows the maize deficit (minimum maize demand for sufficient calorie intake minus domestic production) for Lesotho. Lesotho has seen a large, nonlinear, trend in maize deficit over the last decades (Fig. 1g, black line), driven by increasing population, soil erosion, limited agricultural expansion (only 13% of the country is arable land), poor land-use practices, decreasing soil fertility, and the high number of HIV/AIDS infections that reduces the labour supply^14,19–21^. This has increased the reliance on South Africa for maize imports to meet its national cereal requirement. Precipitation is highly correlated between South Africa and Lesotho (ρ = 0.94, 1979–2018), making production anomalies in both countries also correlated (ρ = 0.38, 1981–2013), although this has changed over the years (S1 and Fig. S1b). The fact that Lesotho and South Africa are in close proximity to each other, and hence subject to the same meteorological conditions and possible future changes in the climate system, makes synchronous maize failures likely to happen in this region now and continuously in the future.

In 2007, the maize production was reduced by 40% in Lesotho compared to 2006, whereas in South Africa, 2007 was the second consecutive production failure, with both 2006 and 2007 witnessing production of 31% below average (over the 1981–2013 period). This reduced the maize available for export to Lesotho (~ 2% of production in South Africa), resulting in a 35,500 tonnes of maize shortage in Lesotho^14^. The production of the two substitute crops, sorghum (20% of cropped area) and wheat (10% of cropped area), also decreased by 42% and 4%, respectively, compared to 2006^14,15^, limiting the dietary substitution to alternative cereal crops. Although sorghum is a more drought tolerant crop^14,15,22^, farmers still decide to plant maize during dry years because of dietary preferences for maize and because markets for sorghum are less well developed^19^. On top of the production loss, the maize price in South Africa, which drives maize prices and hence purchasing power in Lesotho, increased by 41% compared to 2006 and 100% compared to 2005 (Fig. 1f). With the vast majority of small scale farmers in Lesotho (~ 60% population) not being self-sufficient, many households are vulnerable to price spikes as they rely on the grain markets to buy maize. In particular, the poorest households, that spend up to 65% of their total expenditure on staple foods^21^, experienced disproportionate impacts^14^. Overall, the combined shortage and price spike caused imminent food insecurity for 400,000 people in Lesotho, approximately 20% of the total population.

### Influence of climate change

We perform a multi-method and multi-model EEA analysis^13,23^ to develop a synthesis assessment on the role of CC in the occurrence of the drought event in both countries and the co-occurrence of it. EEA compares the likelihood of a given extreme weather event occurring in the actual world (ACT) as observed with its likelihood of occurrence in a counterfactual world (NAT) without human influence on the climate system, the ratio between the two likelihoods being the change in probability, or risk ratio (RR), of an event due to CC. We employ a multi-method and multi-model approach to account for both model-related uncertainties and uncertainties related to methodological assumptions^23^. The RRs for each country’s drought are derived using long-term observational data (CRU-TS^24^) as well as a range of climate models (weather@home^25,26^, HadGEM3-A^27^, as well as ETH-CAM4 and MIROC5 from the HAPPI experiment^28^) (see “Methods”). Moreover, for the joint probability of occurrence, we use the four climate models with a sufficient number of simulations to approximate the tail-end of the distribution (weather@home, HadGEM3-A, ETH-CAM4, MIROC5). The joint probability of the 2007 event in Lesotho and South Africa is derived by analysing the bivariate distribution of the drought in these two countries.

**Figure 2:**
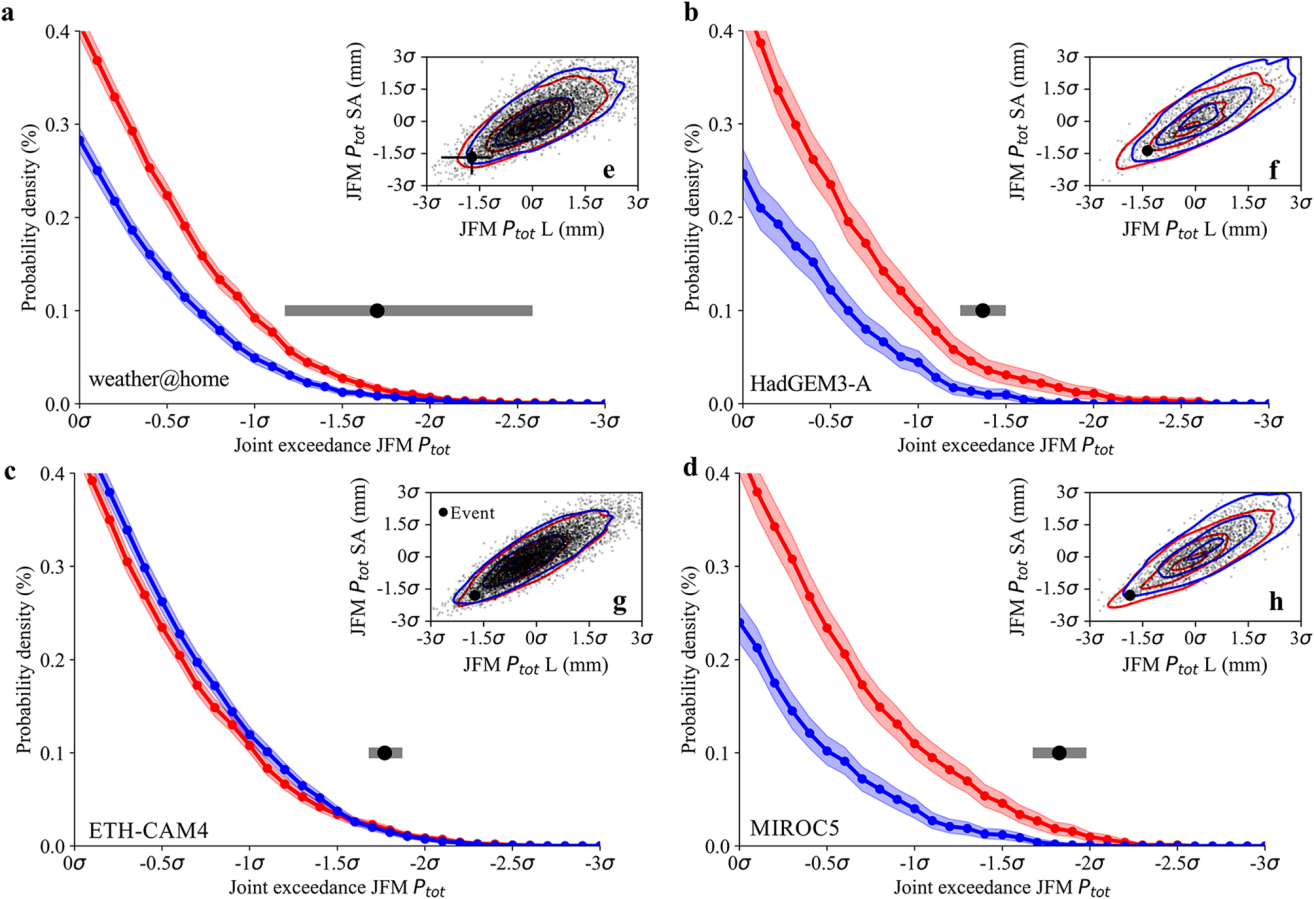
Joint probability plot of the 2007 drought. (**a**) The lower tail (dry–dry) joint probability plot in the weather@home model with red showing the ACT world and blue showing the NAT world without CC. The horizontal grey bars indicate the 10–90% uncertainty estimates of the event using bootstrapping, with the black marker showing the mean estimate. The tails of the distribution are derived empirically. (**b**–**d**) Same as (**a**) but for HadGEM3-A (**b**), ETH-CAM4 (**c**) and MIROC5 (**d**). (**e**) Joint probability plot, with contour lines derived from kernel density estimates, in the weather@home model. The black marker indicates the 2007 event. (**f**–**h**) Same as (**e**) but for HadGEM3-A (**f**), ETH-CAM4 (**g**) and MIROC5 (**h**). The uncertainty around the 2007 event is only visible for weather@home given the larger uncertainty in this model, whereas for the other models the uncertainty is not clearly visible relative to the size of the marker.

We estimate that CC made the 2007 drought event 5.36 (10–90%: 1.51–32.50, Fig. S5a) times more likely in Lesotho and 4.70 (10–90%: 1.53–26.30, Fig. S5b) times more likely in South Africa. The lower tails of the joint exceedance distribution (dry–dry) for the four models are empirically derived and shown in Fig. 2a–d, which are based on the joint distribution functions for the ACT and NAT (Fig. 2e–h). A clear shift can be observed for three out of four models, and overall the likelihood of occurrence of the synchronous drought event increased by a factor 2.14 (10–90%: 1.42–3.16, Fig. S5c) due to CC.

### The role of climate change in food insecurity

We then build a probabilistic model using a set of regression models (see “Methods” for validation) that predicts the production and price anomalies (deviation from detrended time series) based on the precipitation anomalies. We combine this with an approximation of export fraction (percentage of export over production in South Africa) from South Africa to Lesotho (see “Methods”), which varies from ~ 0.9% during normal years to up to 2% during dry years (e.g. 2007). Food availability (< 0 shortage, > 0 surplus) is quantified as the difference between Lesotho’s domestic maize deficit and what is available to import from South Africa. The total imported food value is expressed as the maize deficit in Lesotho and the price of maize in South Africa, which is used as a cumulative indicator of the impacts of prices on household purchasing power. Using the derived RRs, we can construct many plausible counterfactual scenarios of the food security situation without CC which we use to stress-test the system. Stress-testing, referring to exploration of the vulnerabilities of a system based on many plausible scenarios^29^, is used to evaluate the sensitivities of the synchronous maize failures to climate shocks, and its implications for food security in Lesotho.

**Figure 3:**
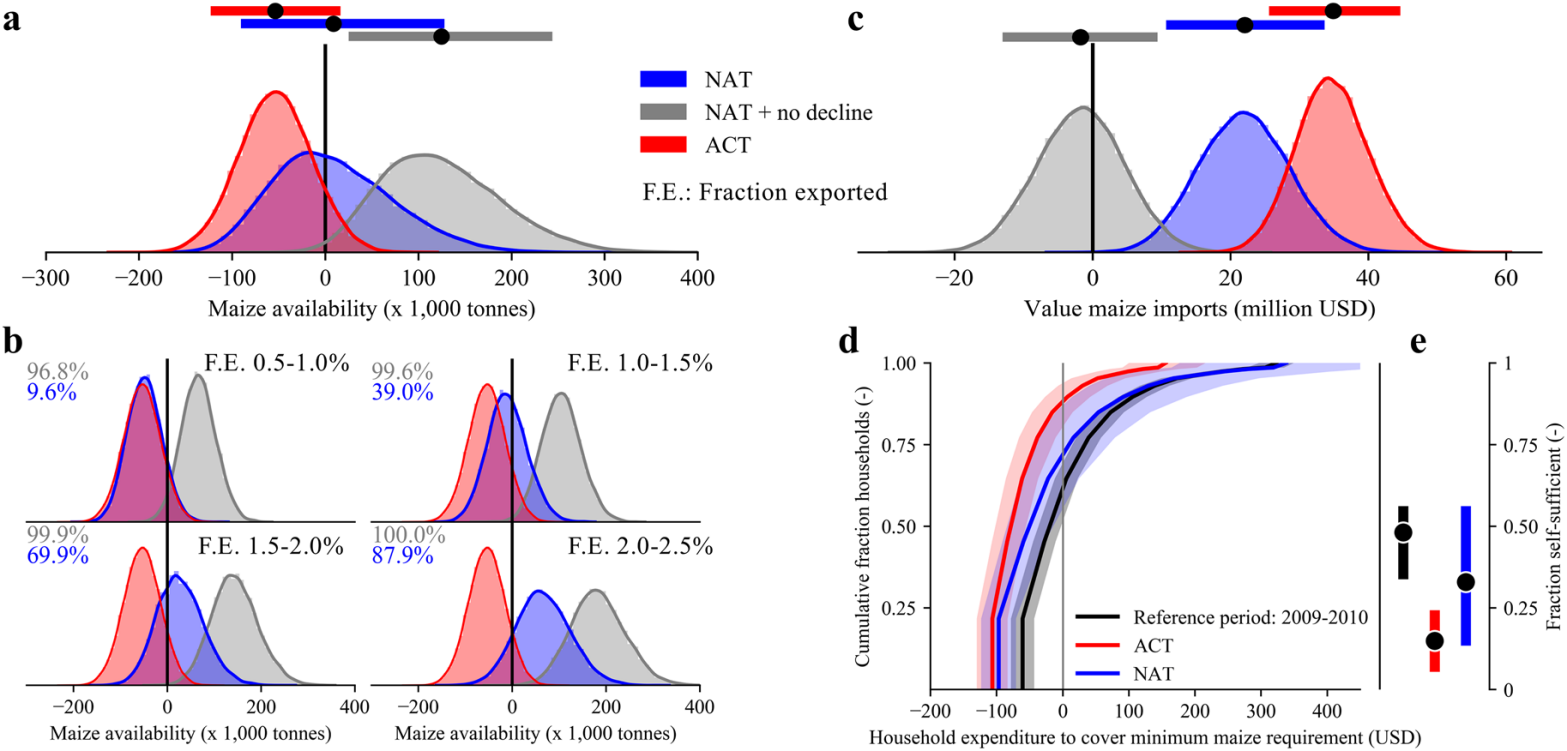
Illustrative plot showing the sensitivity of the food security situation in Lesotho to the 2007-like drought event. (**a**) Probability density plots of the availability of maize to cover minimum consumption needs using the probabilistic model for the ACT (red) and NAT (blue). The horizontal bars at the top illustrate the mean (black marker) and 10–90% uncertainty range of the distribution. The grey distribution plot is the NAT result added to a scenario without no trend in maize deficit in Lesotho. (**b**) A breakdown of plot (**a**) under four different ranges of the fraction of exports (F.E., exports from South Africa to Lesotho over total production South Africa) with the numbers in blue and grey representing the probability of having a positive maize availability value. (**c**) The monetary value of maize imported from South Africa to cover minimum consumption needs in Lesotho. A negative value indicates that the minimum consumption is satisfied, and the surplus maize can be either consumed or sold. (**d**) The cumulative distribution plot of the household expenditure for 13 groups of farming households with varying sizes of farmland operation. The black line is based on the 2009–2010 Agricultural Census of rural households, and the red and blue lines show the model results for the statistical model using the ACT and NAT conditions. The thick line is the mean value and the filled shading the 10–90% uncertainty range based on the 50,000 realizations of the model. (**e**) The fraction of rural households that are self-sufficient in meeting their minimum maize requirement for the reference period (2009–2010), compared to those under ACT and NAT conditions.

We sample 50,000 possible scenarios using the probabilistic model. The spread in the model predictions includes the uncertainties in estimating maize production based on precipitation and, for the NAT simulations, the uncertainties in the fraction of exports from South Africa to Lesotho, which are sampled between observed bounds (0.5–2.5%). Figure 3a shows the results of the probabilistic model, together with the range of the distribution. Our probabilistic model approximates the observed food shortage with a mean of -54,086 tonnes (10–90%: − 119,477 to 11,288 tonnes, observed shortage is within the 68th percentile of the ACT distribution). The NAT distribution plot (blue) has a mean of 8,583 tonnes (10–90%: − 85,802 to 122.760), indicating that a large-scale shortage would have been less likely in a world without anthropogenic CC. For illustrative purposes, we contrast the climate-induced effects to the existing chronic trend of increasing food insecurity in Lesotho and create a hypothetical maize production time series that does not exhibit a declining production trend (NAT + no decline). As can be observed from Fig. 3a, this has a considerably larger effect on the shortage than CC, with a mean of 118,846 tonnes (10–90%: 30,153 to 238,991 tonnes). Figure 3b tracks the probability of maize surplus for four different ranges of fractions of export (F.E.) from South Africa to Lesotho. The probability of maize surplus varies from 9.6 to 87.9% for the NAT simulations and 96.8–100% for the NAT + no decline simulations across the difference F.E.. The distributions of the ACT and NAT in the upper left panel of Fig. 3b (under the F.E. scenario of 0.5–1.0%) are almost identical, indicating that the anthropogenic CC effect is approximately equal to a 1.0–1.5% change in export fraction (compared to the original 2.0% in the ACT). Figure 3b also illustrates the large sensitivity of Lesotho’s food shortage to the amount of production available for export to Lesotho from South Africa. This sensitivity is nonlinear as a less severe drought leads to less crop losses in both countries, and a smaller F.E. need to cover this deficit. Moreover, a less severe drought (less maize loss in Lesotho) also means a higher probability that maize loss can be substituted with available sorghum and/or wheat crops (provided they can fully substitute the sufficient calorie intake). Figure 3c depicts the value of imported maize, which is estimated to be 34.7 million USD (10–90%: 26.2 to 43.9 million USD) in the ACT, 21.9 million USD (10–90%: 11.5 to 32.9 million USD) in the NAT, and -1.7 million USD (10–90%: -12.3 to 8.6 million USD) in the NAT + no decline world (negative value meaning that households would be able to consume or sell the surplus maize). Given that an average rural household spends 175 USD on food and beverages annually^30^, the average per household expenditure on imported maize (total value of imported maize divided by the number of households in 2007) to cover basic food needs constitutes 50.1% (10–90%: 40.4 to 60.2%) in the ACT world and 31.7% (10–90%: 19.6 to 43.8%) in the NAT world. In other words, purchasing power per household is ~ 37% lower in the ACT compared to the NAT. In particular, small-scale farmers would be hit hardest, as their self-sufficiency level is decreased due to lower yield, making them increasingly reliant on food markets (that see higher prices). Using data from the 2009/2010 Agricultural Census of rural households in Lesotho^31^, we approximate the exposure of rural farmers to the combined effect of lower production and price volatility (see “Methods”). 2009/2010 is a relatively neutral year, with no large precipitation, production or price anomaly, and the area of maize planted was almost equal to 2007. Based on the size of the farmland that households operate on (which are aggregated into 13 groups of field sizes), the household size, the yield, and maize price estimates, we evaluate the average household expenditure per group of farmers to cover their minimum consumption requirement. Furthermore, we estimate the aggregated number of households that are self-sufficient, i.e. household production is larger than minimum consumption requirement. As shown in Fig. 3d, the ACT simulations increase the average household expenditure for farmers relative to the reference period. The slope of the ACT relative to the reference period is steeper, indicating that a large share of farmers experienced a non-linear increase in expenditure. This showcases that the majority of rural farmers are affected by a combined effect of production loss and price increase, changing a large number of households from net sellers to net buyers. The number of rural households that are self-sufficient (Fig. 3e) shifts from 48.2% (10–90%: 35.3 to 54.7%) in the reference period to 14.9% (10–90%: 7.0 to 22.8%) in the ACT and 33.0% (10–90%: 15.1 to 54.7%) in the NAT world. Although CC made the drought more severe, which negatively impacted rural households’ self-sufficiency, the NAT world simulations still show a larger number of households that are not self-sufficient relative to the reference period of a relatively neutral year. Therefore, climate variability, whether or not driven by CC, induces strong year-to-year variability in the level of self-sufficiency of rural farmers. The ability to cope with such variations in expenditure to purchase staple foods varies per household and depends on availability of alternative labour income, remittances (main source of income for up to 10–15% of households in some districts^30^), social safety nets and the ability to reduce or change consumption patterns^14,21^.

## Conclusions and policy implications

By combining an extreme event attribution analysis with a probabilistic model of food production and prices, we find that CC increased the likelihood of the 2007 co-occurring drought in South Africa and Lesotho, aggravating the food crisis in Lesotho. However, the effect of CC only reinforces an already chronically upward trend in food shortage in Lesotho that puts the food-supply system of the country in a vulnerable position. In particular, the large climate influence on crop production (due to rainfed agriculture), limited domestic production, and reliance on a climatically connected trading partner contribute to the nonlinear response of the country’s food system to additional climate shocks. Similar system characteristics, such as high import dependency, rain-fed irrigation and large climate variability are observed in other southern African countries^16^.

In light of expected increase in population and a future decline in maize production in Southern Africa due to future CC^4,6,8,10^, our work has several policy implications. First, we highlight that EEA should be extended from a hazard to an impact perspective to truly understand how anthropogenic CC impacts society and identify where and how systems are vulnerable: this type of analysis is much more informative for adaptation decisions than looking at the hazard alone^32^. To do this, both' top-down and bottom-up approaches^33^, combining large-scale climate impact analysis with detailed empirical data on household characteristics and household-level adaptation to drought events, are essential to understand the interaction between the climate and the human-environmental system. From our analysis, it becomes clear that diversification, in terms of cereal production and trading partners, would lower Lesotho’s vulnerability. However, for Lesotho, and many other landlocked countries, access to international grain markets are limited due to high transportation costs. On a local level, reversing the declining production trend can help reduce the dire situation of severe food insecurity (like the 2007 and the more recent 2015/2016 incidents). This may be hard to achieve in the short term, as this trend is ingrained within the wider socio-demographic and environmental characteristics of the country, such as the high poverty and HIV/AIDS rates, the ecological fragile landscape, and limited budget for agricultural investments. Therefore, in the short term, implementing adaptation measures, such as improved drought monitoring, drought-resilient crops and planting strategies, and emergency support and relief mechanisms (e.g. social safety nets, drought insurance)^5,7,16^, are essential to complement longer-term food security strategies. Our stress-testing exercise highlights the potential non-linearities in the regional and local food system, which provides essential information when designing adaptation measures, as significant gains can be made to lower such non-linear impacts to vulnerable households. For instance, the combined effect of social safety nets (to buffer price shocks) and a shift towards drought-resilient crops or irrigation systems should be analysed in an integrated manner to evaluate the combined versus the individual benefits.

Future research should expand on this methodology by applying it across multiple events and to different regions, data availability permitting. In the current work, we only addressed the role of CC in changing the likelihood of a single drought event. Analysing multiple events would strengthen the robustness of the analysis and would help disentangling the different drivers of food insecurity under a range of climatic conditions. For instance, other food insecurity events in the region (e.g., 2015/2016) were due to drought events induced by strong El Niño episodes. Although the role of CC on changes in the frequency and severity of El Niño itself has been documented^35,36^, it remains unclear to what extent CC might cause changes in other aspects, such as food insecurity, propagated through changes in El Niño. Moreover, our methodology can be easily adapted and applied to other regions and crops, including remote production regions that have a climatic teleconnection and are thereby prone to synchronous production failures^34^. Studying other areas and food systems would improve our understanding on the role of CC in changing the likelihood of co-occurring drought events across various spatial scales and help determine the vulnerability of these systems to such events. However, current research efforts are often limited by data limitations (e.g. food supply assessments and trade and household survey data), in particular in data-scarce regions.

Overall, our results suggest that understanding the propagation of CC impacts through trade-dependencies is critical for import-dependent countries that are vulnerable to short-term and chronic food insecurities.

## Methods

### Precipitation and agricultural data

Data on maize production for 1980–2013 is derived from the statistical database of the Food and Agriculture Organization of the United Nations (FAOSTAT^18^). Data on maize prices for South Africa is also available from this source, although only for 1991–2017. Trade data between South Africa and Lesotho for 2010–2018 is taken from UN Comtrade^37^ together with reported production data (to fill in data from 2013 on) in South Africa from the South African Department of Agriculture, Forestry and Fisheries^38^. By dividing the exports over the production in South Africa, the fraction of production available for exporting to Lesotho is calculated. The percentage of production that is exported to Lesotho varies between 0.5% and 1.7% (for 2010–2018), with higher values during years of anomalously low production in South Africa. In 2007, the export fraction was 2%^14^ which was the highest value observed. The per capita maize need is estimated to be 328 g/capita/day^14,39^, which is the minimum maize requirement to meet sufficient calorie intake (2100 cal). This maize need is lower than the annual maize consumption, which is in the order of 167 kg/year during non-drought years. We scale this maize need threshold with the population (based on World Bank data) over the years to get a time series of the annual maize requirement. For 2007, this yields an estimated 240,000 tonnes of maize, which we adopt as total maize demand in Lesotho to meet food security. The maize deficit is defined as total demand minus the domestic production. To define the event threshold, precipitation data is taken from Fifth generation of European Centre for Medium-Range Weather Forecasts (ECMWF) atmospheric reanalyses of the global climate—ERA5, which is chosen because of its higher resolution compared with previous reanalysis precipitation products (0.25°), as well as its improved estimates of the global balance of precipitation and evaporation, and better estimates of precipitation in the deep tropics^17^. Data is averaged over [lon: 27°–29.5°, lat: − 30.5°–− 28.5°] for Lesotho and [lon: 24.5°–30°, lat: − 28–− 26°] for South Africa, over 1979–2018. We compare the ERA5 reanalysis data to more established precipitation products, CRU-TS4.02^24^ and CHIRPS^40^, given that ERA5 is still relatively new. Supplementary Fig. 1a illustrates the co-occurring JFM total precipitation anomalies for Lesotho and South Africa, illustrating that the 2007 event was the most severe on record. Anomalies are calculated by first detrending the time series and then deriving the deviation from the 1979–2018 JFM total precipitation average. Detrending of the precipitation time series is done using a linear regression. Supplementary Fig. 1b shows the production anomalies for both countries. The production time series for South Africa is detrended using a linear regression, whereas for Lesotho we use a locally weighted polynomial regression (lowess) because of the non-linearity in the trend. Overall, the 2007 event was not most severe in synchronous production failure. However, the volatility of production in both countries has decreased over the years (as shown in Fig. 1d,e), indicating improved crop management practices. This has also decreased the correlation between the production anomalies, from ϱ = 0.59 in 1981–1995 to ϱ = 0.21 in 1995–2013. The 2007 synchronous crop failure was, however, very severe if compared to the 1995–2013 period. To derive the price anomalies, we detrend the maize price using a lowess to effectively capture the nonlinear trend in the data.

### Model description and evaluation

We use a set of reanalysis/gridded products and climate models to gain some insights in precipitation in Lesotho and South Africa, given that 2007 was the most severe precipitation deficit in both countries on record. To account for model-related uncertainties, and given that climate modelling in Africa is especially troublesome^41^, we use a diverse set of models from different modelling families: a regional climate model from weather@home^26,42^, a state-of-the-art high resolution global climate model HadGEM3-GA6 model, which is the atmospheric component of the Met Office’s Global Environment Model version 6^27,43^, and two climate models with large ensembles from the ’Half a degree additional warming, projections, prognosis and impacts’^28^ experiment. We picked the two HAPPI models (MIROC5 and ETH-CAM4) that have counterfactual simulations available to represent the world without climate change. Details about the models are included in Supplementary Table 1. See Supplementary Text for the results of the model validation.

### Extreme event attribution

Extreme event attribution seeks to quantify to what extent anthropogenic climate change have altered the probability or magnitude of a particular type of extreme event^13^. The Risk Ratio (RR) of an event is typically used to quantify the role of climate change, which is the ratio of the probability of occurrence (p_1_) of a particular event compared with its probability of occurrence.

(p_0_) had the anthropogenic influences on climate been absent^12,13,44^

1 $$RR = \frac{{p_{1} }}{{p_{0} }}$$

We use both long-term observational data and model outputs to estimates RRs for the events in both countries. We distinguish between the event in the actual world (ACT) and the event in a counterfactual world without human influence (NAT). Using the ERA5 re-analysis data, the JFM total precipitation is found to have a 1-in-40 year return period, and is henceforth used as the event definition (event threshold). The precipitation value corresponding to the 1-in-40 year event is used to find the return period in the modelled world, in order to calculate the RR.

The details of the extreme event attribution and the results for both the single and compound event are included in the Supplementary Information.

### Statistical model

Crop production is driven by a variety of climate, technological and environmental factors, with a large scatter during normal years. However, in dry years, maize growth becomes constraint by soil water availability, affecting processes at the leaf and canopy scale^45^ and consequently production. Maize production in Lesotho and South Africa is mainly rainfed^7^, which results in precipitation during the JFM maize growing season being correlated with maize production (ϱ = 0.69 in South Africa and ϱ = 0.56 in Lesotho). A relationship between precipitation and maize yields is also found in other regions globally, such as Sub-Saharan Africa^46^, the USA^47^ and China^48^.

We create two statistical models that predict crop production anomalies (*C*) using precipitation anomalies (*Pr*) in (*i*) Lesotho and South Africa, respectively. Moreover, we create a model that predicts the maize price (*P*) anomalies for South Africa based on precipitation anomalies. The details of the statistical models adopted are included in Supplementary Information. We can use this model to predict C_i_ in both regions and P in South Africa and transform it back to the original scale. In Lesotho, the deficit (D_L_) is calculated by subtracting the maize demand from C_L_. To get the actual shortage in 2007, after accounting for imports, we use the observed fraction of export (*fe*_*SA*_) in 2007 (2%). The food availability (A_L_) then reads:

2 $${A}_{L,2007}={C}_{SA,2007}*f{e}_{SA,2007}-{D}_{L,2007}$$

Additionally we predict the value of imported food (V_L_) for Lesotho based on the deficit D_L_ and *P*:

3 $${V}_{L,2007}={D}_{L,2007}*{P}_{2007}$$

The model validation is included in the Supplementary Information.

### Probabilistic model food availability and imports

We use an exploratory modelling framework to estimate the influence of climate change in the 2007 event. By sampling our identified drivers (precipitation and export fraction) from a range of possible values, we can explore many possible realizations of the actual and counterfactual world. This can be used to evaluate the sensitivities of the metric in response to different combinations of model input and it can be used to detect critical thresholds for adaptation purposes.

First, using the statistical model we can make predictions of the 2007 food availability and value of imported food in the ACT world. To create realisations of the NAT world, we take a few steps. First, using the extreme event attribution analysis, we found a range of RRs for both Lesotho and South Africa. We sample the RR from a triangular distribution, with the mean values as the most likely value and the 5–95% percentile values as the lower and upper bound. This yield *T*(1.51, 5.36, 32.50) for Lesotho and *T*(1.53, 4.70, 26.30) for South Africa. We fit a GEV distribution to the precipitation data and use this in combination with the RRs to find the precipitation values in a NAT world. We feed these new precipitation values for 2007 in the statistical models and predict the food availability and value of imported food in the NAT world. The export fraction in the NAT is unknown, and as was observed, varies with production and hence rainfall. However, we lack the adequate data coverage to fit a model. Instead we use an uniform distribution between the range of observed fractions, with the lowest observed value as lower bound (0.5%), and the upper bound of 2.0% as observed in 2007. We increase the maximum fraction to 2.5% to account for the possibility of higher fractions. To get the *no decline* time series, we simply filter out the trend in production data in Lesotho. We repeat the procedure described above, but for 2007, the initial maize deficit in Lesotho is considerably less (116,000 tonnes difference). Through Monte Carlo sampling, we run 50,000 plausible scenarios of food availability and import values in a counterfactual world without climate change and/or production decline.

### Household exposure

We look at a first-order proxy of household exposure to the 2007 drought, which depends on the size of the farmland households operate on, the maize yield, the maize consumption to satisfy basic needs, the household size, and the price of maize. We generate two proxies: (1) the percentage of households that are self-sufficient, indicating that the maize harvested on a household level is sufficient to cover the basic consumption needs of maize (120 kg/person/year), and (2) the household expenditure on maize to cover the costs of basic needs in the case that a household is not self-sufficient. In case a household produces more than the basic needs, it can either sell or consume the surplus (which we indicate as a positive value). The details of this model is included in Supplementary Information.

## Supplementary Information

Supplementary Information.

## Data Availability

All data input and codes to analyse the data and reproduce the main findings of the article can be found in Mendeley Data https://doi.org/10.17632/ntjtg6wd5g.1
