# Supplementary material for: Climate change as a driver of food insecurity in the 2007 Lesotho-South Africa drought
Source: Sci Rep. 2021 Feb 16;11:3852. doi: 10.1038/s41598-021-83375-x (PMC7887215; doi:10.1038/s41598-021-83375-x)
Supplement: Supplementary file 1 — Supplementary Information. [file 41598_2021_83375_MOESM1_ESM.docx]

**Supplementary Information**

**Climate change as a driver of food insecurity in the 2007 Lesotho-South Africa drought**

Jasper Verschuur^1^*, Sihan Li^1,2^, Piotr Wolski^3^, Friederike Otto^1^

^1^Environmental Change Institute, University of Oxford, United Kingdom

^2^Oxford E-Research Centre, Department of Engineering Science, University of Oxford, United Kingdom

^3^Climate System Analysis Group, University of Cape Town, South Africa

*[jasper.verschuur@keble.ox.ac.uk](mailto:jasper.verschuur@keble.ox.ac.uk)

**Supplementary Text**

**Climate models**

For model evaluation, we compare all model and reanalysis/gridded data to ERA5. We look at monthly average precipitation values, and compare the long-term seasonality over the years, as shown in Supplementary Fig. 2. For weather@home and HadGEM3-A (1987-2013), a 27 year time frame is considered, whereas for the two HAPPI models only the 10 years included are used (2006-2015).

The different reanalysis products are comparable (ERA5, CRU-TS, CHIRPS), with ERA5 showing higher precipitation values during the boreal winter (DJF), while boreal summer months all show comparable estimates. For South Africa, the HAPPI models tend to overestimate DJF precipitation, while weather@home and HadGEM3-A better capture the seasonality. For Lesotho, all climate models overestimate DJF precipitation, and ERA5 has higher precipitation values than CRU-TS and CHIRPS. This is to be expected given the differences in resolution, i.e. lower resolution products do not capture the high precipitation driven by elevation gradients that are present in Lesotho. Second, we compare the ability of the climate models to capture the correlation of the precipitation time series between the two regions. The models show a lower correlation than ERA5 (Supplementary Table 2), but these correlation values are usually considered good over the tropics area.

Third, we directly compare the monthly precipitation values over the time frame considered (see above), using the root mean square error (RMSE) and mean absolute error (MAE) as goodness of fit criteria. For the climate models, we use the ensemble mean time series. The results for South Africa and Lesotho are shown in Supplementary Fig. 3 and 4, respectively. CRU-TS and CHIRPS tend to have a good fit, with a slight underestimation of the precipitation values. The weather@home model is marginally better than the HadGEM3-A model, although for South Africa the weather@home model does not capture high precipitation intensities, whereas the HadGEM3-A model does. The ETH-CAM4 and MIROC5 models tend to overestimate monthly precipitation, but do perform well for Lesotho, in particular MIROC5.

**Extreme Event Attribution**

Using CRU-TS4.02 data, we derive the RR directly from the observational time series, following the method described in Van Oldenborgh et al.^1^. First, we fit a linear regression between global mean surface temperature (GMST) anomalies and JFM total precipitation between 1901-2017, for both South Africa and Lesotho. Using this fitted relationship between GMST and JFM precipitation, we can filter out the trend and derive a NAT time series of precipitation. CRU-TS is used instead of ERA5 because of its long record length. For South Africa, we use a ’Generalised Extreme Value’ (GEV) distribution to fit the data, whereas for Lesotho a Gamma fit is found to more accurately fit the data. Using this, we find the RRs, and create uncertainty bounds using bootstrapping (10,000 times). For the HadGEM3-A and weather@home models, we compare ACT and NAT runs directly. For HadGEM3-A, we use the ensemble mean for both ACT and NAT to best capture the precipitation and fit a GEV distribution to the data. To get uncertainty bounds, we bootstrap from all ensemble members (10,000 times). For weather@home we follow a similar procedure by fitting a GEV to the ensemble mean, and to get an uncertainty bound of the RRs, we bootstrap by sampling different combinations (ACT vs NAT) of ensemble members. For the MIROC5 and ETH-CAM4 models from HAPPI experiment, 10 years of data is available with a large number of ensembles. We can assume stationarity in the time series (given it’s only 10 years), allowing us to treat these 10 year time series as independent and therefore as different alternative climatologies. We lump all the data from the 10 years into one data pool in order to empirically derive the return periods for both the ACT and NAT scenarios. Bootstrapping (10,000 times) is used to derive confidence intervals.

To estimate the RRs for the compound event (co-occurring drought event in Lesotho and South Africa), only the climate models are used (weather@home, HadGEM3-A, ETH-CAM4 and MIROC5). In order to calculate the joint probability, we fit the joint precipitation data to a bivariate Gaussian distribution. Although exceedance probability could be derived empirically, the Gaussian distribution ensures robustness in approximating the tails, and shows a good overall agreement with the empirical distribution data (low Kolmogorov-Smirnov (KS) statistic between the standardized data and a standard normal distribution using a KS-test). We calculate the RR by finding the exceedance probabilities in the ACT and NAT joint distribution plots, and create uncertainty bounds from the uncertainty around the precipitation values. These bounds are derived using the bootstrapping results from the univariate case.

Supplementary Fig. 5 shows the results of the analysis from the various data sources. Supplementary Fig. 5a-b show the result for individual drought event in South Africa and Lesotho, clearly demonstrating that the events have become more frequent under anthropogenic climate change. Data is skewed towards higher RRs, indicating that taking the uncertainty into account is essential here. The mean values are coherent for the different methods and models used, while uncertainties differ. To get a synthesis result, we simply averaged all models and techniques (both mean and uncertainty). Even though this is simplistic, there is no concrete rationale to weight a model or technique more than the other. RRs for the 2007 event are estimated to be 5.36 (90%: 1.51 – 32.50) for Lesotho and 4.70 (90% 1.53 – 26.30) for South Africa. Supplementary Fig. 4c shows the result for the compound event, with coherence between the weather@home, HadGEM3-A and MIROC5 model (bigger RR), but an opposite results for ETH-CAM5 (less likely). The synthesis result show that the event has a RR of 2.14 (90%: 1.42 – 3.16).

**Statistical model**

As mentioned we fit both production models and a price model. For precipitation, we include the current year precipitation and two years (prior) of lag (*t*−1, *t*−2) to account for soil moisture memories from previous months, and to account for multi-year variability of soil moisture related to El Nino cycles in this region^2^. Moreover, we include a dummy variable () to indicate whether or not year *t* and year *t*−1 are dry years (drier than the climatology period). This is included to account for the consideration that production becomes constrained by precipitation in dry years and thereby changes the production-precipitation relationship, and thereby the price changes. In summary, production anomalies are a function of:

$C_{t,i} = f(Pr_{t,i},Pr_{t-1,i},Pr_{t-2,i},Xd_{t,i},Xd_{t-1,i}$) (1)

and similarly for price anomalies:

$P_{t} = f(Pr_{t},Pr_{t-1},Pr_{t-2},Xd_{t},Xd_{t-1}$) (2)

A second order polynomial fit yields the best fit to the data. We expect a second order function between precipitation and production, since in dry years production will decrease, whereas production will peak during normal years. During wet years, excessive precipitation could also reduce production: previous work showed that excessive rainfall can have a similar effect on production as precipitation deficits^3^. Apart from precipitation anomalies, maize production is also affected by temperature anomalies^4^. High temperatures both increase water demand of crops while lowering water supply due to larger evapotranspiration, which has a strong influence on water stress^5,6^. We don’t explicitly include temperature in our modelling framework (for multicollinearity reasons, as temperature and precipitation are correlated in this region), but since precipitation deficits are associated with higher than normal temperatures, the effect is implicitly included in the statistical model.

Supplementary Fig. 6 shows the observed and predicted values for the maize production anomalies in South Africa (a), the maize deficit anomalies in Lesotho (b), and the price anomaly in South Africa (c). The models performs relatively well for production in South Africa (: 0.60), production in Lesotho (: 0.52) and price in South Africa (: 0.75). The values during drier years are better predicted (closer to the 1:1 line) than during wetter years, which is the part we are interested in. The 2007 event is also predicted well in both countries (red marker in Supplementary Fig. 6)

**Household exposure model**

We base our exposure data on the 2009/2010 Lesotho Agricultural Census of rural households^7^, which contains information about the rural farming households in Lesotho (which we use as a reference year). The 2009/2010 agricultural season was comparable to an average agricultural year, with no drought or price spike during this year. In addition, a similar amount of maize was planted in this year compared to 2006/2007 (151,717 ha in 2009/2010 versus 156,500 ha in 2006/2007). The rural farming household population was estimated to be 217,748 households, of which the majority of households are small-scale farmers (65% of households operate on less than 1.5ha). We generate two proxies: 1) the percentage of households that are self-sufficient, indicating that the maize harvested on a household level is sufficient to cover the basic consumption needs of maize (120 kg/person/year), and 2) the household expenditure on maize to cover the costs of basic needs in the case that a household is not self-sufficient. In case a household produces more than the basic needs, it can either sell or consume the surplus (which we indicate as a positive value).

We take the household distribution per area of farmland from^7^, where households are classified into 13 groups according to the size of the land they operated on (001-0.49 till 6+ ha). The yield in 2009/2010 was equal to 0.9 tonnes/ha^7^ and the average rural household size is 4.7 people^8^. The household size may vary between groups, and the land of operation is specified within a range of 0.5ha instead of a single value. Therefore, we construct 50,000 combinations of realizations of farmland that is operated on, and households size per group that are in line with the survey statistics. We sample the farmland and household size from an uniform distribution function, with bounds equal to the upper and lower bound for farmland (e.g. between 0.01 and 0.49) and between 3.5 and 5.9 (range of between-district variability in household size) for household size. The cumulative farmland that is operated on is combined with the yield estimate (0.9 t/ha), and a correction factor (that specifies which share of the farmland operation is used for maize) is added in order to make the total maize production in line with the 2009/2010 production (151-413 tonnes). We do the same for the household size sampling, by adding a correction factor to make sure the average household size is equal to 4.7. By multiplying the household size with the maize need, we can estimate whether or not the household production is sufficient to meet the basic maize needs. In order to calculate the household expenditure, we subtract the household production from the basic maize need (either deficit or surplus) and multiply this with the 2009/2010 price of maize, which is based on the South African maize price. A positive value here (surplus) indicates that a household can either sell or consume the surplus maize. Most households will consume the surplus maize, given that the average maize consumption is 40% more than the minimum maize need (167 kg/capita/year versus 120 kg/capita/year).

Using the ACT and NAT data for 2007, we do a similar exercise, but now for changing maize prices and yield. We do not look at the NAT+no decline scenario, given that it is hard to attribute how the no decline scenario would have changed the household size, farmland size and yield. The 2007 yield estimate was equal to 0.4 t/ha^9^, which we assign to the mean of the ACT simulations of production in Lesotho. Using the 50,000 realizations (as mentioned before) of ACT and NAT productions in Lesotho, we estimate the yield per simulation (by scaling it with the ACT mean) and derive the percentage of households that are self-sufficient per simulation. Additionally, we calculate the average household expenditure per group based on the combined yield and price estimates per realization. We do not account for any changes in consumption patterns or take into account that households may switch from maize to another non-cereal product. However, given that the other cereal substitutes were similarly impacted by the drought, switching to another cereal crops is unlikely. Moreover, as indicated by^10^, the cross-elasticity of maize in Lesotho is low in case of price increases, indicating that switching to other (non-cereal) food products is not likely (also given the lower calorie intake of for example vegetables compared to cereals).

**Supplementary Figures**

**Supplementary Fig. 1. Precipitation and production anomalies Lesotho and South Africa.** (a) Joint occurrence of JFM total precipitation anomalies in Lesotho and South Africa. Anomalies are found after detrending the time series and calculating the deviation from the long-term average (1979-2018). (b) Production anomalies of maize in Lesotho and South Africa. Note the different units on the x-axis and y-axis.

**Supplementary Fig. 2. Validation seasonality Lesotho and South Africa.** (a) Seasonal mean and 10-90% uncertainty based on the interannual variability for all years. (b) Same as (a) but for Lesotho.

**Supplementary Fig. 3. Climate model validation South Africa.** (a) Comparison between monthly average precipitation from ERA5 and that from CRU-TS 4.02 (in mm/month) in South Africa. (b,c,d,e,f) same as (a) but for CHIRPS (b), weather@home (c), HadGEM3-A (d), ETH-CAM4 (e) and MIROC5 (d).

**Supplementary Fig. 4. Climate model validation Lesotho**. Same as Fig. S3, but for Lesotho.

**Supplementary Fig. 5. Results risk ratios.** (a) Results of the risk ratio for the extreme event in South Africa, showing the individual results (mean + uncertainty) and synthesis. RR>1 implies event is becoming more likely in the ACT compared with the NAT (b) Same as (a) but for Lesotho (c) Same as (a) but for the joint probability of the compound event.

**
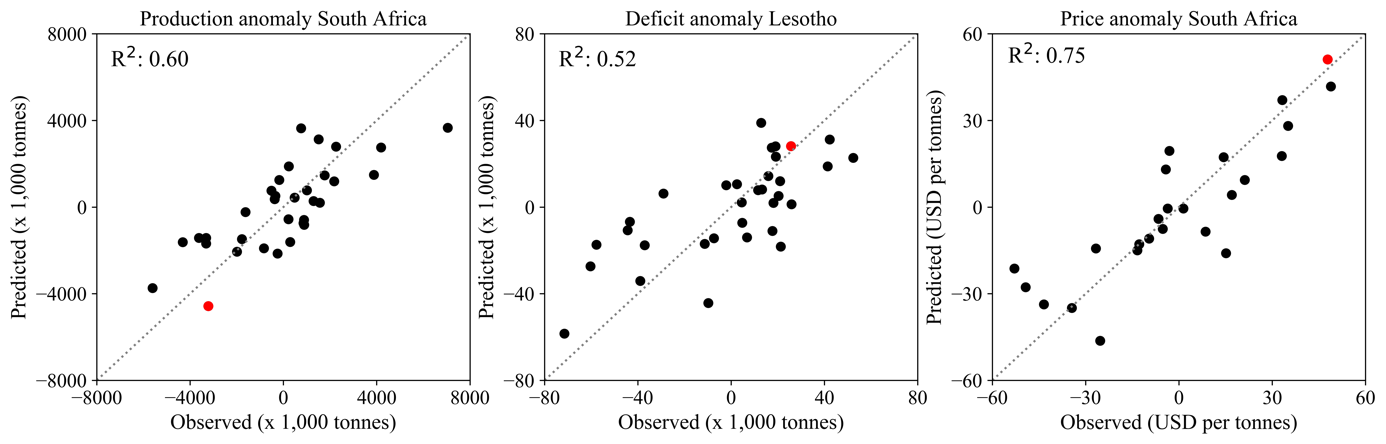
**

**Supplementary Fig. 6. Model validation statistical model**. (a) Error plot of the predicted and observed production anomalies in South Africa (b) Same as in panel (a), but for Lesotho (c) Error plot of the predicted and observed maize price anomalies in South Africa. The dotted line in each panel indicates the 1-1 line.

**Supplementary Tables**

| **Model** | **Reference** | **Time frame** |
| --- | --- | --- |
| weather@home: the Met Office Hadley Centre regional model HadRM3P | Allen et al. (1999) | Historical: 1987-2013 |
| (or PRECIS) implemented over South Africa, generated through | Massey et al. (2015) |  |
| weather@home within climateprediction.net | Guillod et al. (2018) |  |
| HadGEM-A: HadGEM3-GA6 model, the atmospheric component of the | Walters et al. (2017) | Historical: 1987-2013 |
| Met Office’s Global Environment Model version 6 | Ciavarella et al. (2018) |  |
| ETH-CAM4 from HAPPI: NCAR-DOE Community Atmosphere Model | Neale et al. (2013) | Historical: 2006-2015 |
| version 4 (CAM4) coupled to the Community Land Model version4 (CLM4)) |  |  |
| MIROC5 from HAPPI: contributed by National Institute for Environmental | Shiogama et al. (2013) | Historical:2006-2015 |
| Studies, Tsukuba, Japan |  |  |

**Supplementary Table 1.** Information of models used in this study, time frame considered, and pertinent references.

| ERA5 | weather@home | HadGEM3-A | ETH-CAM4 | MIROC5 |
| --- | --- | --- | --- | --- |
| 0.91 | 0.71 (0.60 - 0.84) | 0.80 (0.74 - 0.84) | 0.85 | 0.83 |

**Supplementary Table** **2.** Comparison of the Pearson correlation coefficient of the precipitation between the two regions. For weather@home/HadGEM3-A, uncertainty bounds are derived from the ensemble members.

**References**

1. van Oldenborgh, G. J., van Urk, A. & Allen, M. The absence of a role of climate change in the 2011 Thailand floods. *Bull. Am. Meteorol. Soc.* **93**, 1047–1049 (2012).

2. Miralles, D. G. *et al.* El Niño-La Niña cycle and recent trends in continental evaporation. *Nat. Clim. Chang.* **4**, 122–126 (2014).

3. Li, Y., Guan, K., Schnitkey, G. D., DeLucia, E. & Peng, B. Excessive rainfall leads to maize yield loss of a comparable magnitude to extreme drought in the United States. *Glob. Chang. Biol.* **25**, 2325–2337 (2019).

4. Lobell, D. B., Bänziger, M., Magorokosho, C. & Vivek, B. Nonlinear heat effects on African maize as evidenced by historical yield trials. *Nat. Clim. Chang.* **1**, 42–45 (2011).

5. Schlenker, W. & Lobell, D. B. Robust negative impacts of climate change on African agriculture. *Environ. Res. Lett.* **5**, (2010).

6. Lobell, D. B. *et al.* The critical role of extreme heat for maize production in the United States. *Nat. Clim. Chang.* **3**, 497–501 (2013).

7. Bureau of Statistics. *2009/2010 Lesotho Agricultural Census: Rural households and crop statistics*. **1**, (2012).

8. Bureau of Statistics Lesotho. 2006 Lesotho population and housing census analytical report, Volume IIIB Socio Economics Characteristics. **III**, (2006).

9. FAO. *FAO/WFP Crop and food supply assessment mission to Lesotho*. (2007).

10. FAO. Impact of food prices increase among Lesotho’s poorest. 1–28 (2016).
